# Supplementary material for: Transcriptomic Insights into Mechanisms of Early Seed Maturation in the Garden Pea (Pisum sativum L.)
Source: Cells. 2020 Mar 23;9(3):779. doi: 10.3390/cells9030779 (PMC7140803; doi:10.3390/cells9030779)
Supplement: Supplementary file 1 [file cells-09-00779-s001.zip › Supplementary materials/FileS4.zip!/FileS3/TFs - Results/Summary Sheets - PATs/PAT report.pdf]

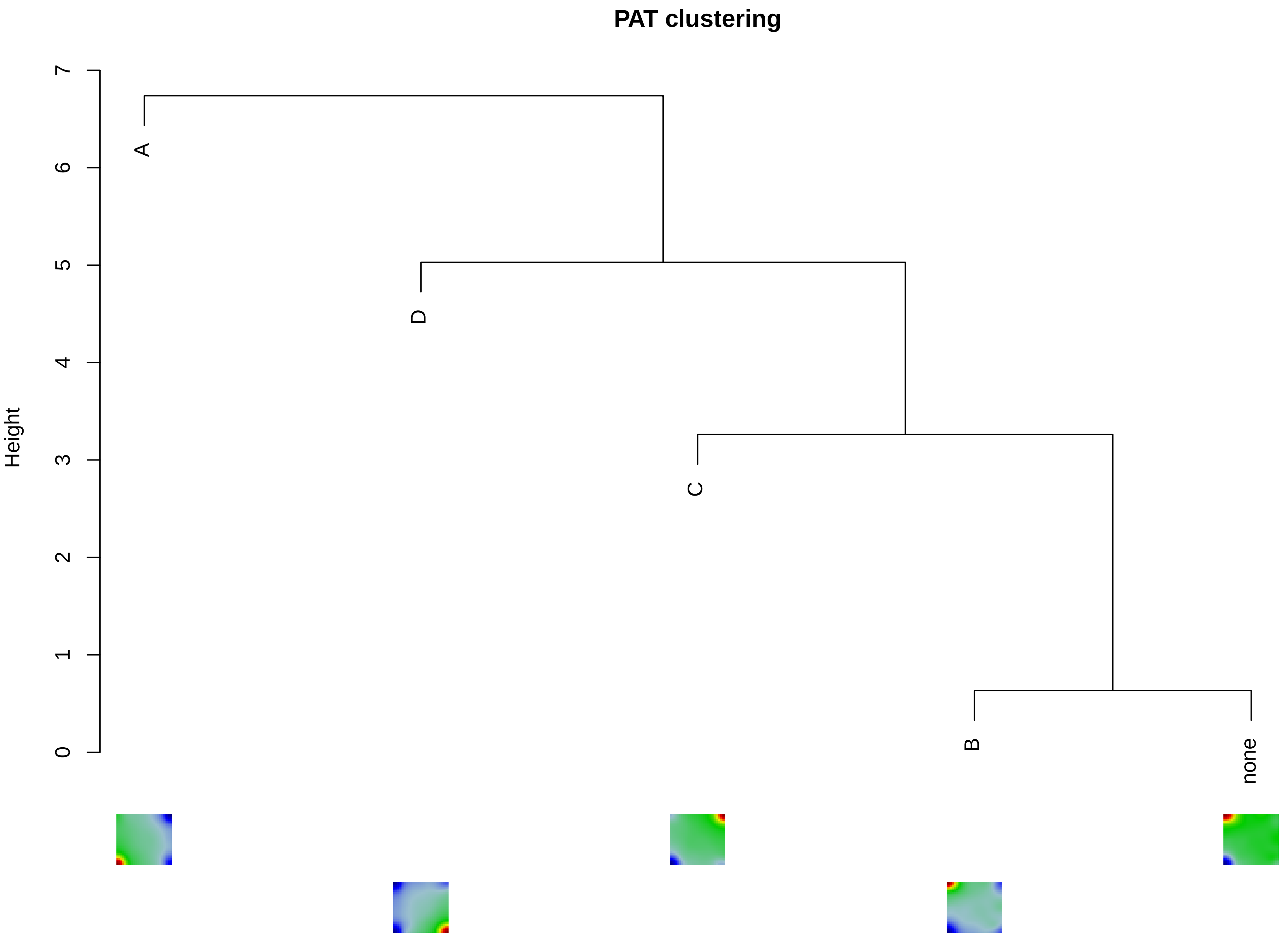
PAT clustering
Height
0
1
2
3
4
5
6
7
A
D
C
B
none

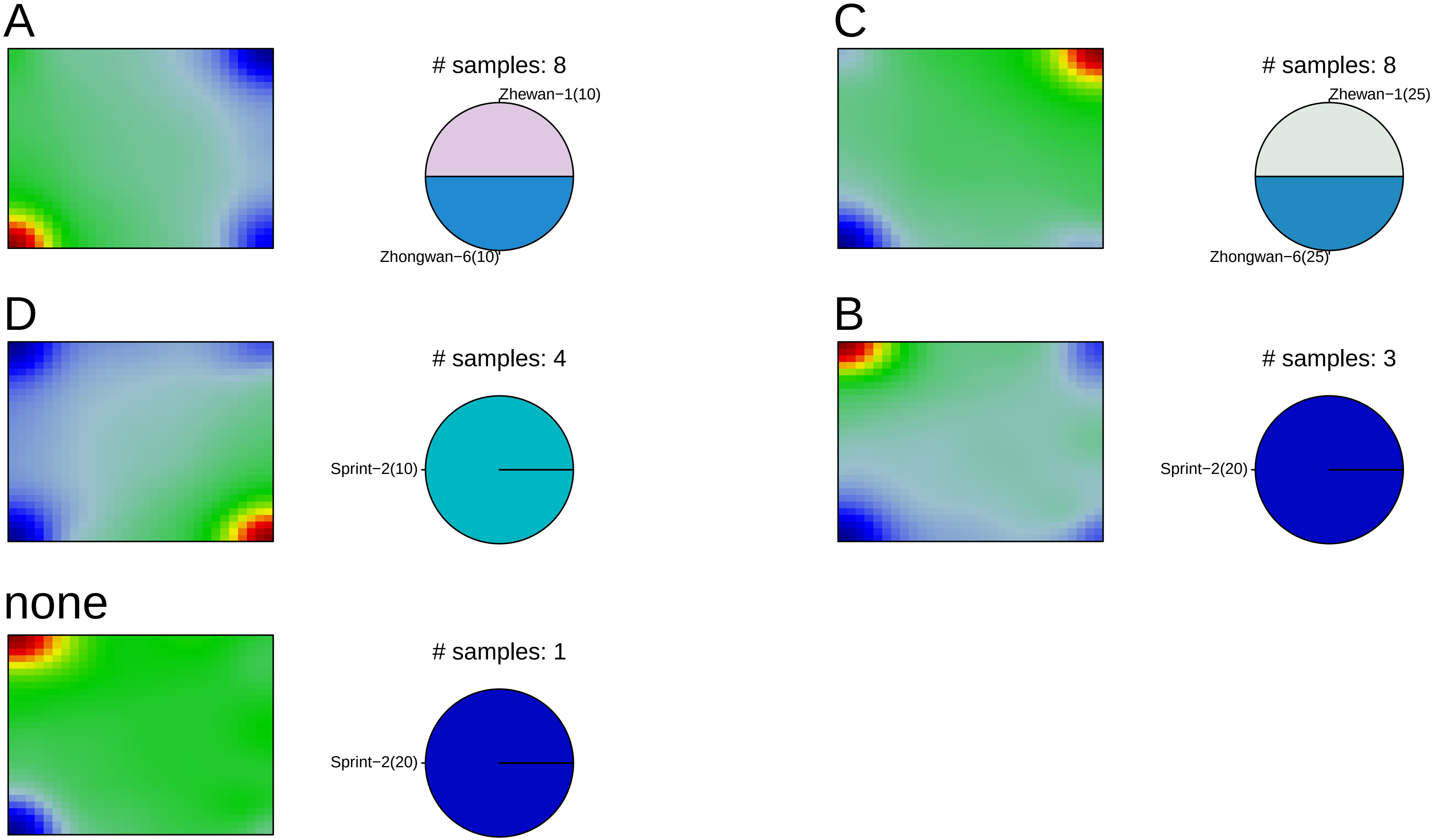
A
samples: 8
Zhewan−1(10)
Zhongwan−6(10)
C
samples: 8
Zhewan−1(25)
Zhongwan−6(25)
D
samples: 4
Sprint−2(10)
B
samples: 3
Sprint−2(20)
none
samples: 1
Sprint−2(20)

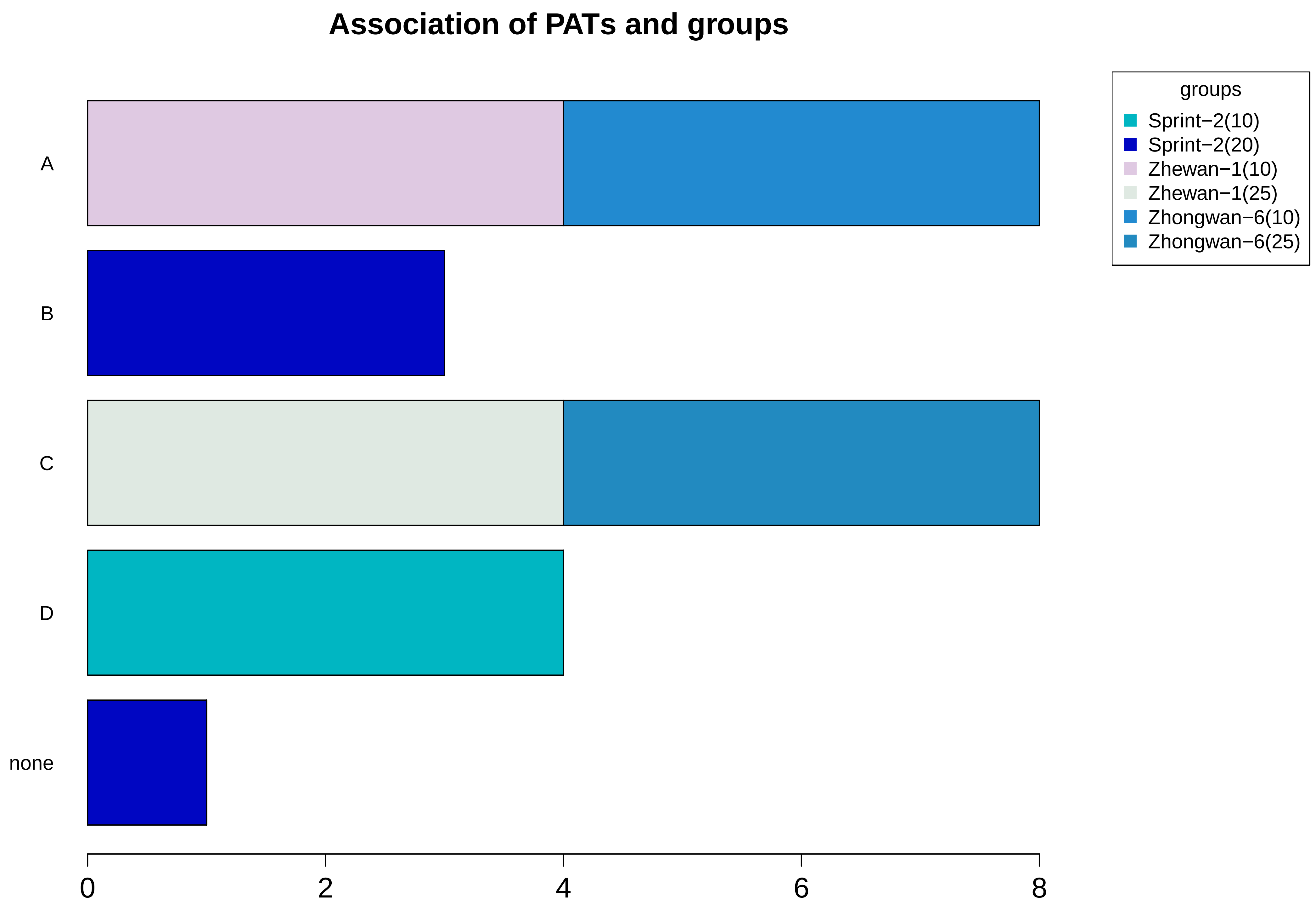
Association of PATs and groups
A
B
C
D
none
0
2
4
6
8
groups
Sprint−2(10)
Sprint−2(20)
Zhewan−1(10)
Zhewan−1(25)
Zhongwan−6(10)
Zhongwan−6(25)

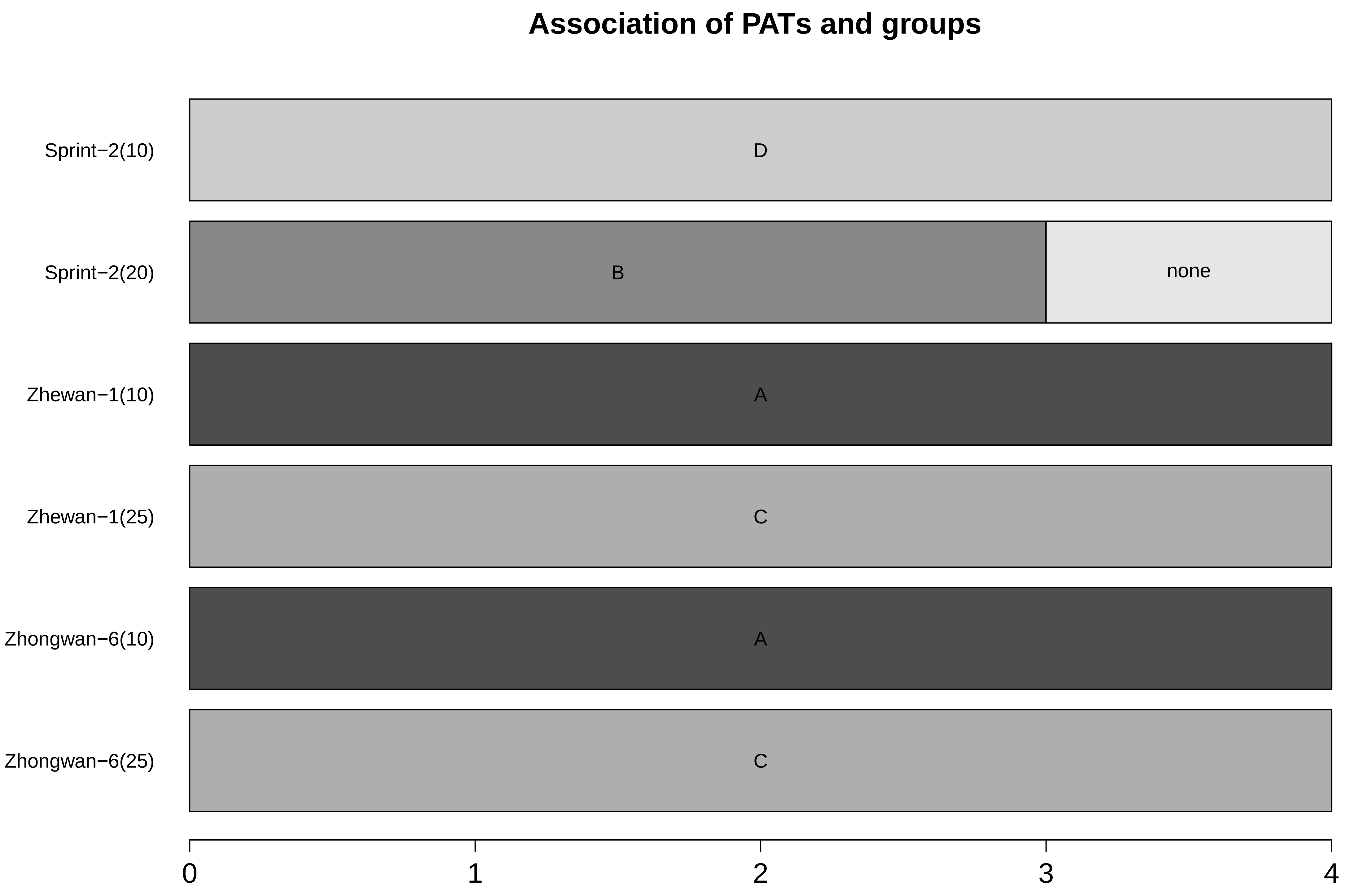
Association of PATs and groups
Sprint−2(10)
Sprint−2(20)
Zhewan−1(10)
Zhewan−1(25)
Zhongwan−6(10)
Zhongwan−6(25)
D
B
none
A
C
A
C
0
1
2
3
4
